# Concomitant *KIT/BRAF* and *PDGFRA/BRAF* mutations are rare events in gastrointestinal stromal tumors

**DOI:** 10.18632/oncotarget.8768

**Published:** 2016-04-16

**Authors:** Sabrina Rossi, Marta Sbaraglia, Marta Campo Dell'Orto, Daniela Gasparotto, Matilde Cacciatore, Elena Boscato, Valentina Carraro, Luisa Toffolatti, Giovanna Gallina, Monia Niero, Emanuela Pilozzi, Alessandra Mandolesi, Fausto Sessa, Aurelio Sonzogni, Cristina Mancini, Guido Mazzoleni, Salvatore Romeo, Roberta Maestro, Angelo P. Dei Tos

**Affiliations:** ^1^ Department of Pathology and Molecular Genetics, Treviso General Hospital, Treviso, Italy; ^2^ Department of Experimental Oncology, CRO, Aviano, Italy; ^3^ Department of Clinical and Molecular Medicine, University of Rome “La Sapienza”, Rome, Italy; ^4^ Department of Pathology, University of Marche, Ancona School of Medicine, Ancona, Italy; ^5^ Department of Pathology, Macchi Fondation, Varese, Italy; ^6^ Department of Pathology, General Hospital, Bergamo, Italy; ^7^ Department of Pathology, Azienda Ospedaliera-Universitaria, Parma, Italy; ^8^ Department of Pathology, General Hospital, Bolzano, Italy

**Keywords:** GIST, BRAF-mutated GIST, KIT/BRAF concomitant mutations, Imatinib resistance, BRAF VE1 antibody, Pathology Section

## Abstract

**AIM:**

The *BRAF* mutation is a rare pathogenetic alternative to *KIT/PDGFRA* mutation in GIST and causes Imatinib resistance. A recent description of *KIT* and *BRAF* mutations co-occurring in an untreated GIST has challenged the concept of their being mutually exclusive and may account for *ab initio* resistance to Imatinib, even in the presence of Imatinib-sensitive *KIT* mutations. *BRAF* sequencing is generally limited to *KIT/PDGFRA* wild-type cases. Hence, the frequency of concomitant mutations may be underestimated.

**METHODS:**

We screened for *KIT* (exon 9, 11, 13, 17), *PDGFRA* (exon 12,14, 18) and *BRAF* (exon 15) mutations a series of 407 GIST. Additionally, we evaluated the BRAF V600E mutation-specific antibody, VE1, as a surrogate for V600E mutation, on a series of 313 GIST (24 on whole sections, 288 cases on tissue array), including 6 cases molecularly ascertained to carry the BRAF V600E mutation.

**RESULTS:**

No concomitant *KIT*/*BRAF* or *PDGFRA*/*BRAF* mutations were detected. *BRAF* mutation was detected only in one case, wild-type for *KIT/PDGFRA.* All the 6 *BRAF*-mutant cases stained positive with the VE1 antibody. A weak VE1 expression was observed in 14/287 (4.9%) *BRAF* wild-type cases, as observed also in 2/6 *BRAF*-mutant cases. Overall in our series, sensitivity and specificity of the VE1 antobody were 100% and 95.1%, respectively.

**CONCLUSION:**

The concomitance of *BRAF* mutation with either *KIT* or *PDGFRA* mutation is rare in GIST. In these tumors, moderate/strong VE1 immunoreactivity is a valuable surrogate for molecular analysis. Instead, genotyping is warranted in the presence of weak VE1 staining.

## INTRODUCTION

BRAF is a serine/threonine protein kinase of the RAF family and belongs to the RAS-RAF-MEK-ERK signalling pathway, which leads to the activation of several cytoplasmic and nuclear targets with transcriptional function, e.g. ETS11, c-JUN and c-MYC. This signalling pathway is triggered by several receptor tyrosine kinases (TKs) such as KIT and PDGFRA. In human cancer the RAS-RAF-MEK-ERK effector pathway is commonly activated, often with gain-of-function mutations in either RAS or RAF gene family members [1].

*BRAF* mutations have been found in a wide range of tumors (almost 7% of all cancers), both benign (melanocytic nevi [2], intestinal hyperplastic polyps, sessile serrated polyps/adenomas [3], gangliogliomas and pilocytic astrocytomas [4]), and malignant (hairy cell leukemia [5], melanomas [6], pleomorphic xanthoastrocytomas [4], papillary thyroid carcinomas [7], serous ovarian tumors [8], biliary tract carcinomas [9], colon adenocarcinomas [10, 11], lung adenocarcinomas [12], seminomas [13], mastocytosis [14] and gastroenteropancreatic neuroendocrine tumors [15]).

The *BRAF* mutation has also been reported in a small subset of Gastrointestinal Stromal Tumors (GIST) [16].

GIST are the most common mesenchymal tumor of the gastrointestinal tract [16]. Around 85% of sporadic primary GIST harbor activating mutations in either the *KIT* (65%) or *PDGFRA* gene (20%) [17], both encoding type III RTKs, and are variably sensitive to RTK-inhibitors, mainly Imatinib. The remaining cases (about 15%) represent a heterogeneous group of tumors that generally do not respond to Imatinib and include pediatric GIST, SDH-deficient GIST, NF1-associated GIST, and GIST driven by mutations downstream the TK pathway, e.g. BRAF [16]. The *BRAF* mutation is a rare event in primary GIST. About 8% of the cases devoid of *KIT/PDGFRA* mutations bear the *BRAF* mutation [17–21]. Although the use of next generation sequencing (NGS) mutation panels is gaining ground in the clinical diagnostic setting, in the majority of pathology laboratories molecular diagnosis still relies on Sanger sequencing and in most centers the *BRAF* mutation is investigated after ruling out the most common *KIT* and *PDGFRA* mutations. Hence, the frequency of this “alternative mechanism” and its co-existence with *KIT/PDGFRA* mutations is likely underestimated. *BRAF*-mutated tumors are morphologically and phenotypically indistinguishable from “classical” GIST. However, location-wise, they seem to cluster in the small bowel [17–21]. About 50% of the BRAF-mutated GIST reported so far fall in the AFIP high-risk category [17–21]. Nevertheless, their rarity and the lack of follow-up data in most series leaves uncertainty as to the correlation between pathologic risk assessment and actual clinical behavior (See Table 1).

**Table 1:** BRAF-mutated GIST in the literature

N°	Ref.	Age/Sex	Primary/ Relapsed	Site	Size (cm)	Morph.	Mit./ 50HPF	AFIP Risk
**1**	Agaram	52/F	Primary	Sm. Int.	10	Mixed	90	HR
**2**	Agaram	55/F	Primary	Sm. Int.	10	Spindle	5	LR
**3**	Agaram	49/F	Primary	Sm. Int.	9	Mixed	50	HR
**4**	Agaram	66/M	Relapsed**	Perit.	NA	Rhabdo	NA	NA
**5**	Agaimy	70/M	Primary	Stom.	0.4	Spindle	<5	NR
**6**	Agaimy	80/M	Primary	Sm. Int.	0.4	Spindle	<5	NR
**7**	Hostein	53/M	Primary	Sm. Int.	20	Spindle	6	HR
**8**	Hostein	38/M	Primary	Sm. Int.	2.5	Mixed	5	IR
**9**	Hostein	63/M	Primary	Stom.	2.5	Spindle	NA	NA
**10**	Hostein	78/M	Primary	Stom.	NA	Spindle	1	LR
**11**	Hostein	51/F	Primary	Sm. Int.	3	Spindle	10	HR
**12**	Hostein	58/M	Primary	Duod.	2.5	Mixed	1	IR
**13**	Hostein	58/M	Primary	Sm. Int.	2.5	Spindle	6	IR
**14**	Hostein	41/M	Primary	Sm. Int.	2.5	Spindle	3	LR
**15**	Hostein	50/F	Primary	Perit.	2.8	Epith.	50	HR
**16**	Miranda	NA	Primary	Sm. Int.	NA	NA	NA	HR
**17**	Miranda	NA	Primary	NA	NA	NA	NA	NA
**18**	Falchook	60 M	Primary	NA	15	Spindle	6*	NA
**19**	Zheng	75 M	Relapsed**	Perit.	NA	Rhabdo	8	NA
**20**	Rossi	69/M	Primary	Sm. Int.	4.6	Spindle	4	LR
**21**	Rossi	36/F	Primary	Sm. Int.	8.5	Mixed	3	UR
**22**	Rossi	66/F	Primary	Sm. Int.	5.4	Mixed Polym.	8	HR
**23**	Rossi	63/M	Primary	Sm. Int.	11.2	Mixed	12	HR
**24**	Rossi	42/F	Primary	Sm. Int.	3.8	Spindle	7	HR
**25**	Rossi (current)	89/F	Primary	Sm. Int.	1.8	Spindle	1	NR

Sm. Int.= Small Intestine; Stom.= Stomach; Duod.= Duodenum; Perit.= Peritoneum; Rhabdo= Rhabdomyoblastic differentiation; Polym.= Polymorphic; HR= High Risk; IR= Intermediate Risk; LR= Low Risk; NR= No Risk; UR= Unknown Risk;

* In this case the number of mitoses was counted on 10 HPF;

** Tumor developed under imatinib therapy;

While the prognostic role of BRAF is still being debated, its predictive value in response to therapy is well documented. BRAF encodes a kinase molecule downstream of the TK pathways and its mutation constitutively activates the cascade, thereby bypassing the inhibitory effects of Imatinib. The *BRAF* mutation causes both *ab initio* resistance to imatinib treatment [19, 22] and secondary resistance when it occurs as a secondary event in *KIT/PDGFRA*-mutated GIST relapsing under therapy [19, 23]. Recently, a case of GIST with dual *BRAF* and *KIT* mutations has been reported in an untreated patient [21], challenging the concept of *KIT/PDGFRA* and *BRAF* mutation being mutually exclusive in primary GIST. These authors suggested that the concomitance of *KIT* and *BRAF* mutations might explain the resistance phenomena observed in a fraction of GIST carrying Imatinib-sensitive mutations (about 5%).

These combined data prompted us to perform a comprehensive evaluation of the involvement of BRAF kinase in GIST development and progression. To this end, we screened a series of 407 GIST cases referred to Treviso General Hospital. In addition, we sought to address the accuracy of immunohistochemistry-based screening to detect *BRAF* mutation as a surrogate for molecular analysis using BRAF V600E mutation-specific antibody VE1. This reagent has shown good sensitivity and specificity in detecting V600E-mutated cells in most, although not all, of the investigated tumor types [6, 24–49] [50] (See Supplementary Table 1).

## RESULTS

Results from *KIT/PDGFRA/BRAF* molecular analysis are summarized in Table 2. Only one out of the 407 cases proved to carry a *BRAF* mutation. This case, a small intestinal untreated GIST, was devoid of *KIT* or *PDGFRA* mutations. No case of concomitant *KIT* and *BRAF* or *PDGFRA* and *BRAF* mutations was found, not even in relapsed cases. Conversely, six concomitant *KIT/KIT* and one concomitant *KIT/PDGFRA* mutations were detected in seven metastases that developed under Imatinib treatment (six peritoneal and one hepatic) (Table 3). This supports the notion that BRAF activation compensates for lack of TK mutation in GIST but does not seem to play a relevant role in secondary resistance, where *KIT* exon 13 and exon 17 mutations seem to be prevalent, in line with published data [16]. In addition, a double mutation was found in a localized untreated rectal GIST; in this case, both mutations involved *KIT* exon 11 (Lys558Gln and Val560del).

**Table 2:** Frequency of *KIT/PDGFRA/BRAF* mutations in 407 GIST cases

Gene	Exon	Cases (N)	Cases (%)
KIT	11	243	59.7
KIT	9	39	9.6
KIT	13	11	2.7
KIT	17	3	0.8
PDGFRA	18	37	9.1
PDGFRA	12	6	1.5
PDGFRA	14	4	1
BRAF	15	1	0.2
KIT/PDGFRA/BRAF WT	-	55	13.5
KIT/KIT	11/11	1	0.2
KIT/KIT	11/13	2*	0.5
KIT/KIT	11/17	3*	0.8
KIT/KIT	13/17	1*	0.2
KIT/PDGFRA	13/18	1*	0.2
Total	-	407	100.0

* Imatinib-treated GIST with double mutations

**Table 3:** Cases with secondary mutations developed under Imatinib therapy

	Primary tumor			Metastasis		
	Site	Gene/exon	Mutation	Site	Gene/exon	Mutation
1	NA	KIT/11	Met552_Pro573delinsIle	abdominal cavity	KIT/17	Tyr823Asp
2	stomach	KIT/11	Trp557_Glu561del	abdominal cavity	KIT/13	Val654Ala
3	NA	KIT/11	Asn566_Pro573del	abdominal cavity	KIT/17	Asn822Lys
4	duodenum	KIT/11	Val560_Leu576del	Liver	KIT/13	Met651Ile
5	NA	KIT/11	Glu556_Val560delinsHis	abdominal cavity	KIT/17	Asn822Lys
6	stomach	KIT/13	Lys642Glu	abdominal cavity	KIT/17	Asn822Lys
7	stomach	KIT/13	Lys642Glu	abdominal cavity	PDGFRA/18	Arg841_Asp842delinsLys

**Figure 1:**
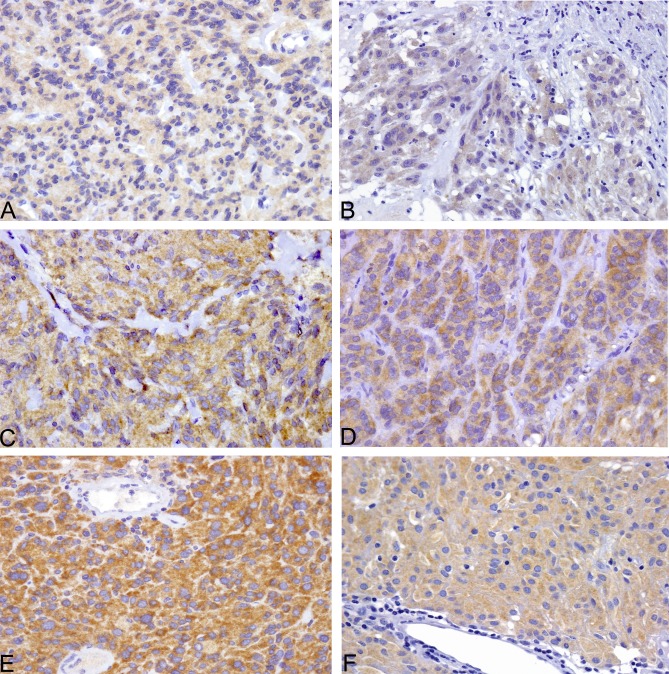
All six BRAF V600E-mutated GIST of the series were VE1 positive, with weak cytoplasmic staining in two cases (A,B), moderate staining in two cases (C, F) and strong staining in two cases (D, E)

As for the immunohistochemical results, VE1 antibody yielded weak, non-specific, diffuse cytoplasmic staining in the normal gastric/intestinal epithelium and muscularis propria. No nuclear reactivity was observed. A clear-cut positive pattern, with fine granular cytoplasmic accumulation was evident in all four *BRAF*-mutated control GIST and the single V600E-mutated case included in our series. This reactivity was weak in two cases, moderate in one and strong in two cases (Figure 1A-1E), with a prevalent homogeneous pattern, but for one case where it was patchy. No reactivity was observed in *BRAF* wild-type cases. Overall, there was complete agreement in this study series (21 cases) between BRAF V600E molecular analysis and IHC, with a sensitivity and specificity of 100% (Table 4).

**Table 4:** VE1 sensitivity and specificity in the first and second study set

	Whole section set	TMA set
**Cases**	25	288
**BRAF-mutated cases**	5	1
**BRAF-mutated VE1-positive cases** **(% sensitivity)**	5 (100)	1 (100)
**BRAF-WT VE1-positive cases** **(% specificity)**	0 (100)	14 (95.1)

Sensitivity = 100.00 % (95% CI: 16.55 % to 100.00 %)

Specificity = 95.12 % (95% CI: 91.95 % to 97.31 %)

To corroborate this initial finding, a second set of 288 GIST, belonging to a population-based study and arranged in tissue arrays, was also analysed. The single *BRAF*-mutant case included in this series turned out to be positive for VE1, with moderate granular staining of the cytoplasm (Fig. 1 F). Two hundred and seventy-three cases were clearly negative. Conversely, weak cytoplasmic-positive staining was observed in 14 cases, three of which belonged to the *KIT/PDGFRA/BRAF* wild-type subgroup, seven to the *KIT*-mutated and four to the *PDGFRA*-mutated subgroup. Negativity for *BRAF* mutation was double checked in these cases. In this second study set, the concordance rate between BRAF V600E molecular analysis and IHC was thus 95.1%, with a sensitivity of 100% and specificity of 95.1% (Table 4). Importantly, none of the *BRAF*-wild-type GIST showed moderate/strong staining.

## DISCUSSION

*BRAF* mutation has been reported in a small subset of primary *KIT* and *PDGFRA* wild-type GIST [17–21] and in rare relapsed cases receiving Imatinib therapy [19, 23]. To date, 22 BRAF-mutated cases have been described, but the actual role of V600E mutation in GIST pathobiology is far from being defined. Except for a few studies [17, 18, 20, 21], *BRAF* status is usually investigated in *KIT/PDGFRA* mutation-negative cases only.

To investigate the relevance of concomitant *BRAF* and *KIT/PDGFRA* mutations in primary and secondary resistance to Imatinib, we conducted a molecular study of the hot spots of *KIT, PDGFRA* and *BRAF*. Only one case out of the 407 analyzed GIST carried the BRAF V600E mutation. This case was wild-type for *KIT/PDGFRA*. No concomitant mutations were found in *KIT* and *BRAF* or *PDGFRA* and *BRAF*. We detected only one double mutation in a localized/untreated context, consisting of a point mutation and a deletion affecting nearby nucleotides in *KIT* exon 11, similarly to our previous report [51]. Secondary mutations in *KIT*-mutated cases involved the more classical tyrosine kinase domains of KIT (exon 13 and 17) in six out of seven cases. Intriguingly, we also found one gastric GIST with a primary *KIT* exon 13 mutation (Lys642Glu) which relapsed three years later under Imatinib, with a secondary mutation in *PDGFRA* exon 18 (Arg841_Asp842delinsLys). To the best of our knowledge, this is the second case reported so far of acquired resistance involving a different kinase from the one affected by the primary mutation [52]. Our results support the notion that the *BRAF* mutation plays a minor role as a concomitant alteration in both primary and secondary resistance.

In order to optimize the method for detecting *BRAF*-mutated cases in GIST, we evaluated the expression of the mutation-specific antibody VE1 in a large series of GIST and compared the results with direct sequencing of *BRAF* exon 15. Our findings indicate that VE1 antibody is highly sensitive for the presence of *BRAF* V600E mutation in GIST, as all six BRAF-mutated cases scored VE1 positive with moderate/strong staining intensity in four cases. Moderate/strong staining was detected exclusively in *BRAF*-mutated GIST, whereas none of the *BRAF*-mutation negative cases displayed such intensity. These findings indicate that significantly intense VE1 staining reliably predicts the presence of *BRAF* mutation. This is in line with a recent study on a series of 38 GIST, in which VE1 strong expression was limited to the 2 *BRAF*-mutant cases included [53]. Additionally, in that series, a weak VE1 staining was found only in a fraction of the *BRAF*-mutation negative cases [53]. Differently, in our series, 2 out of 6 *BRAF*-mutant cases showed only a weak VE1 staining, as well as 14 out of 287 (4.9%) *BRAF* wild-type cases. Hence, our results should caution the pathologist to interpret as either negative or positive for *BRAF*-mutation those GIST that show a weak VE1 expression. Instead, we consider that in presence of a weak staining, the molecular assessment of *BRAF* gene status is highly recommended.

Although the frequency of the *BRAF* mutation in GIST is limited (<1%), the presence of this mutation has a high impact on patients' management. If on one hand it causes resistance to TK-inhibitors [18, 22], on the other it sensitizes the tumor to BRAF inhibitors. Falchook *et al*. recently reported a GIST case effectively treated with Dabrafenib [22]. NGS is gaining ground in the diagnostic setting, thus allowing for the simultaneous assessment of a wider set of molecular biomarkers. While waiting for this approach to be fully implemented by pathology laboratories, we believe that at the present time VE1 immunostaining may represent a valuable tool to address BRAF mutation status in GIST.

## MATERIALS AND METHODS

### Tumor samples

To investigate the role of *BRAF* mutation in GIST, *KIT* (exon 9, 11, 13, 17), *PDGFRA* (exon 12, 14, 18) and *BRAF* (exon 15) mutations were sequenced in a series of 407 cases (including 358 personal consultation cases referred to one of the authors [ADT], and 49 in-house cases) from 398 patients. Eight patients had multiple GIST either in the context of neurofibromatosis (three cases) or in a non-syndromic context (five cases) [54]. Informed consent was obtained from all living patients. Two hundred and fifteen were men and 183 were women. Age ranged between 24 and 91 years (median 63). Clinical records were available for 344 of the 398 patients. Two hundred and eighty-seven out of 353 tumors were primary GIST: 262 located in the gastrointestinal tract (142 gastric, 17 duodenal, 88 from the small intestine, one from the colon, 14 from the sigma-rectum) and 23 extra-gastrointestinal (12 from the abdominal cavity and pelvis, and nine from the retro-peritoneum). The remaining 68 cases were either relapsed or metastatic GIST.

The median size of the primary tumors was 5.5 cm (range 0.5 to 35 cm) and the median mitotic index was 4/50 HPF (range 0 to 180). The risk category could be determined in 221 cases on the basis of the AFIP classification (17 no risk, 13 very low, 61 low, 49 intermediate and 81 high risk tumors).

The predictive value of VE1 staining, as a surrogate for BRAF mutation analysis, could be assessed by immunohistochemistry on a subset of 21 (in-house) cases. Four GIST carrying the BRAF V600E mutation were retrieved from a previous series [17] and included as positive controls.

A further series of 288 cases, arranged in 25 tissue microarrays (TMA) deriving from a large population-based Italian study [17] [55], was also tested for VE1. Each tumour was represented by two to four cores in each array. In this study set, *KIT/PDGFRA/BRAF* status had previously been determined by sequential screening of the different exons until the mutation was detected, in the following order: *KIT* exon 11, *KIT* exon 9, *PDGFRA* exon 18, *PDGFRA* exons 12 and 14, *KIT* exons 13 and 17. Cases devoid of *KIT/PDGFRA* mutation were further investigated for BRAF V600 mutations [17]. Of these 288 cases, one was B*RAF*-mutated (V600E), 188 were *KIT*-mutated (165 with exon 11, 18 with exon 9, three with exon 13 and two with exon 17 mutation), 61 were *PDGFRA*-mutated (49 with exon 18, five with exon 14, six with exon 12 mutation), one case carried a double *KIT* mutation (Asn659Asp and Pro567Leu), and 38 were wild-type. Three of the 165 *KIT* exon 11 mutations were homozygous.

### Molecular analyses

DNA was extracted from representative blocks of formalin-fixed/paraffin-embedded tissues with tumor cellularity greater than 80%. 10-μm-thick sections were deparaffinized by serial xylene/ethanol washings. DNA was extracted using the EZ1 Biorobot (Qiagen GmbH). KIT, PDGFRA and BRAF mutation analysis was performed by PCR and Sanger sequencing using the ABI PRISM 3100 Genetic Analyzer (Applied Biosystems), as previously described [17] [51] [54].

### Immunohistochemistry

Immunohistochemistry (IHC) was performed on freshly-cut 3-μm-thick, paraffin-embedded tissue sections, using VE1 antibody specific for BRAF V600E mutation (clone VE1, Spring Bioscence, Pleasanton, CA). Only cases for which the block was available to provide freshly cut sections were included in the study. To optimize the method, a range of conditions were tested, in relation to both antibody dilution (1:25, 1:50, 1:100) and antigen retrieval (pH6 and pH9 buffer). A series of 10 melanomas, eight with V600E mutation and two with alternative BRAF mutations (V600K, K601E), were used as positive and negative control cases, respectively.

The selected protocol included heat-induced epitope retrieval (PTLINK Dako) with high pH (pH9) and antigen-antibody reaction at 1:100 dilution for 40 minutes (KIT ENVISION FLEX, Dako) in an automated immunostainer (Dako Autostainer, DakoCytomation, Glostrup, Denmark). For four BRAF-mutated cases, VE1 antibody was also tested on whole sections which had been cut seven years earlier. Notably, the intensity of the staining was much weaker than the results obtained on the freshly-cut sections, highlighting that the time gap between cutting and staining highly impacts on VE1 performance.

All immunostained slides were evaluated with blinding to clinical, histopathologic and genetic data by three histopathologists of varying experience. Interobserver agreement was high with approximately 10% of the cases re-reviewed collegially. Where there was any disagreement, the sections were re-reviewed and a consensus opinion reached.

Tumors were considered as positive when the tumor cells showed weak, moderate or strong cytoplasmic staining, and as negative when the tumor cells showed either faint cytoplasmic staining or no staining.

## SUPPLEMENTARY MATERIAL TABLE


