# Supplementary material for: Concomitant KIT/BRAF and PDGFRA/BRAF mutations are rare events in gastrointestinal stromal tumors
Source: Oncotarget. 2016 Apr 16;7(21):30109–18. doi: 10.18632/oncotarget.8768 (PMC5058667; doi:10.18632/oncotarget.8768)
Supplement: Supplementary file 1 [file oncotarget-07-30109-s001.pdf]

Concomitant *KIT/BRAF* and *PDGFRA/BRAF* mutations are rare events in gastrointestinal stromal tumors

Supplementary Material

Supplementary Table 1. VE1 studies reported in the literature

	Number of cases	Tumor type studied for BRAF V600E mutation	Sensitivity (%)	Specificity (%)
Skorokhold, 2012	29	Melanoma	87.5	100
Long, 2013	97	Melanoma	97	98
Busam, 2013	64	Melanoma	100	100
Boursault, 2013	230	Melanoma	97.3	100
Colomba, 2013	103	Melanoma	100	100
Marin, 2014	67	Melanoma	90-93	97-100
Koperek, 2012	144	Thyroid papillary carcinoma	100	-
Zagzag, 2013	37	Thyroid papillary carcinoma	89	100
Zimmermann, 2013	48	Thyroid papillary carcinoma	93.8	93.8
Crescenzi, 2014	30	Thyroid papillary carcinoma	100	100
Na, 2015	104	Thyroid papillary carcinoma	100	94
Ilie, 2013	450	Lung adenocarcinoma	90.5	100
Sasaki, 2013	26	Lung adenocarcinoma	100	95.2
Ida, 2013	37	Pleomorphic xantoastrocytoma	100	100
Bösmüller, 2012	179	Ovarian serous tumors	100	100
Capper, 2012	85	Brain metastasis	High*	High*
Routhier, 2013	152	Melanoma, Thyroid papillary K, Lung K, gliomas	98	97
Adackapara, 2013	52	Colon adenocarcinoma	71 (35**)	74 (100**)
Sinicrope, 2013	75	Colon adenocarcinoma	100	100
Capper, 2013	91	Colon adenocarcinoma	100	98.75
Toon, 2013	201	Colon adenocarcinoma	99.5	100
Affolter, 2013	31	Colon adenocarcinoma	100	100
Kuan, 2013	128	Colon adenocarcinoma	100	94.5
Sajanti, 2014	147	Colon adenocarcinoma	100	99.3

Lasota, 2014	113	Colon adenocarcinoma	85	68
Piton, 2015	30	Colon adenocarcinoma	100	100
Schafroth, 2015	485	Colon adenocarcinoma	92.9-93.3	100
Sperveslage, 2013	78	Pituitary adenoma	0	0

* Concordance of 97.1%   ** If staining is only moderate or strong, they are considered positive
